# Intraspecific genetic diversity of the fish-infecting microsporidian parasite *Pseudokabatana alburnus* (Microsporidia)

**DOI:** 10.3389/fmicb.2023.1129136

**Published:** 2023-03-09

**Authors:** Meiqi Weng, Xintong Zhang, Zhaozhe Xin, Sijia Xue, Qianqian Zhang, Aihua Li, Jinyong Zhang

**Affiliations:** ^1^Laboratory of Aquatic Parasitology and Microbial Resources, School of Marine Science and Engineering, Qingdao Agricultural University, Qingdao, China; ^2^Key Laboratory of Aquaculture Diseases Control, Ministry of Agriculture and State Key Laboratory of Freshwater Ecology and Biotechnology, Institute of Hydrobiology, Chinese Academy of Sciences, Wuhan, China; ^3^College of Advanced Agricultural Sciences, University of Chinese Academy of Sciences, Beijing, China; ^4^Laboratory for Marine Biology and Biotechnology, Pilot National Laboratory for Marine Science and Technology, Qingdao, Shandong, China

**Keywords:** microsporidia, Rpb1, genetic recombination, sexual reproduction, genetic variation

## Abstract

*Pseudokabatana alburnus* is a xenoma-forming fish microsporidium, firstly described from the liver of the *Culter alburnus* from Poyang Lake in China. In the present study, *P. alburnus* was firstly reported from the ovary of 6 other East Asian minnows, including *Squaliobarbus curriculus*, *Hemiculter leucisculus*, *Cultrichthys erythropterus*, *Pseudolaubuca engraulis*, *Toxabramis swinhonis*, and *Elopichthys bambusa*. Genetic analysis revealed high sequence diversity in the ribosomal internal transcribed spacer region (ITS) and the largest subunit of RNA polymerase II (Rpb1) loci of *P. alburnus* isolated from different hosts and locations. The variation of Rpb1 mainly occurred in the 1,477–1737 bp regions. The presence of a wide variety of Rpb1 haplotypes within a single fish host, together with evidence of genetic recombination suggested that *P. alburnus* may have the intergenomic variation and sexual reproduction might be present in other hosts (possibly freshwater shrimp). Phylogenetic analysis and population genetic analysis showed that there was no geographical population divergence for *P. alburnus*. Homogeneity and high variability of ITS sequences indicates that ITS may be a suitable molecular marker to distinguish different *P. alburnus* isolates. Our data confirm the broad geographical distribution and host range of *P. alburnus* in the middle and lower reaches of the Yangtze River. Additionally, we emendated the genus *Pseudokabatana* to exclude the infection site, liver as one of the taxonomic criteria, and proposed that fish ovary was be the general infection site of *P. alburnus*.

## 1. Introduction

Microsporidia are obligate intracellular eukaryotic parasites that have been reported in nearly all animal taxa, ranging from protists to vertebrates including humans (Stentiford et al., 2013b; Cali and Takvorian, 2014). Microsporidia are characterized by the production of resistant spores possessing the evaginable polar tube which transmit the infective sporoplasm to the host cytoplasm or nucleoplasm during the spore germination (Dussaubat et al., 2013; Roudel et al., 2013; Vavra and Lukes, 2013). Over 1,600 microsporidia belonging to about 200 genera have been described worldwide, among which more than 160 species falling within 22 genera are known to infect fishes (Liu et al., 2019). Some fish-infecting microsporidians are economically important pathogens for wild and aquaculture fish, such as *Glugea pagri* infecting the red sea bream *Pagrus major* (Su et al., 2014), *Enterospora epienpheli* infecting the juvenile groupers *Epinephelus* spp. (Xu et al., 2017), and *G. plecoglossi* infecting the ayu *Plecoglossus altivelis* (Zhou et al., 2018).

The early classification of microsporidia was mainly based on spore morphological characteristics and ultrastructural features of the life developmental stages. However, due to the wide occurrence of high plasticity of life cycles and spore morphology of some species, the traditional taxonomy of Microsporidia is increasingly challenged by the small subunit ribosomal RNA (SSU rDNA) sequences based molecular phylogeny. Therefore, a comprehensive taxonomic approach of integrating morphological characters, ultrastructural data, host, and molecular phylogeny of based on SSU rDNA sequence data has been reached a consensus for the classification of the microsporidia (Stentiford et al., 2013a,b; Weng et al., 2021b). Generally, the SSU rDNA sequence are highly similar in fish-infecting microsporidia and cannot provide sufficient resolution to fully resolve the phylogenetic relationships between closely related species (Nilsen et al., 1998; Cheney et al., 2000). The ribosomal internal transcribed spacer region (ITS) and the large subunit ribosomal RNA (LSU rDNA) has been increasingly employed to resolve this issue for they possess sufficient variable loci (Bell et al., 2001; Lom and Nilsen, 2003). Furthermore, referring to ITS and LSU sequences comparison, intraspecific genetic variation was identified in *Ovipleistophora diplostomuri* and several *Loma* species (Brown et al., 2010; Weng et al., 2021a). If this intraspecific genetic variation is linked with possible phenotypic changes remains unknown, however, intraspecific differences in ultrastructure of spores was previously found in *Pleistophora hyphessobryconis* (Li et al., 2012). The switch of host or infected tissue represent important driving factors for genetic differentiation, even novel speciation. Meanwhile, genetic variation of pathogens is possible associated with the virulence of pathogens which directly determine the infection consequences (Eggert et al., 2021; Shaw et al., 2021). Therefore, insights into population genetics of some microsporidia species of economic importance will benefit for the control of microsporidiosis in aquaculture. Although most individual microsporidian species solely infect one or two closely related hosts, 2% were reported to infect multiple host species (Muraeanu et al., 2021). So, some genetically related hosts-infecting species will be ideal models to explore the evolutionary biology of Microsporidia at population scale.

For the occurrence of xenoma formation, different infection sites and independent phylogenetic branch, we previously erected the genus *Pseudokabatana* to discriminate the teleost skeletal muscle-infecting genus *Kabatana*, with *P. alburnus* as the type species which infects the liver of an East Asian minnow *Culter alburnus* (Xenocypridiae, Cypriniformes) collected from Lake Poyang, the biggest freshwater lake in China (Liu et al., 2019). Here, *P. alburnus* were firstly identified and characterized from the ovary of various East Asian minnow species, including *Squaliobarbus curriculus*, *Hemiculter leucisculus*, *Cultrichthys erythropterus*, *Pseudolaubuca engraulis*, *Toxabramis swinhonis*, and *Elopichthys bambusa* which were sampled from various geographical locations in the middle and lower reaches of the Yangtze River. Furthermore, the intraspecific genetic variations (between-hosts and between-host locations) of *P. alburnus* were investigated based on the sequence comparisons of ITS and Rpb1 which were widely employed molecular markers for population genetics of microsporidia (Maside et al., 2015; Gonzalez-Tortuero et al., 2016). Results showed that high nucleotide diversity was observed in the ITS and Rpb1 of *P. alburnus* isolated from different hosts and locations. The present work unequivocally deepens our insights into the reproduction, tissue tropism, and life cycle of this fish-infecting microsporidian.

## 2. Materials and methods

### 2.1. Sample collections

**Figure 1:**
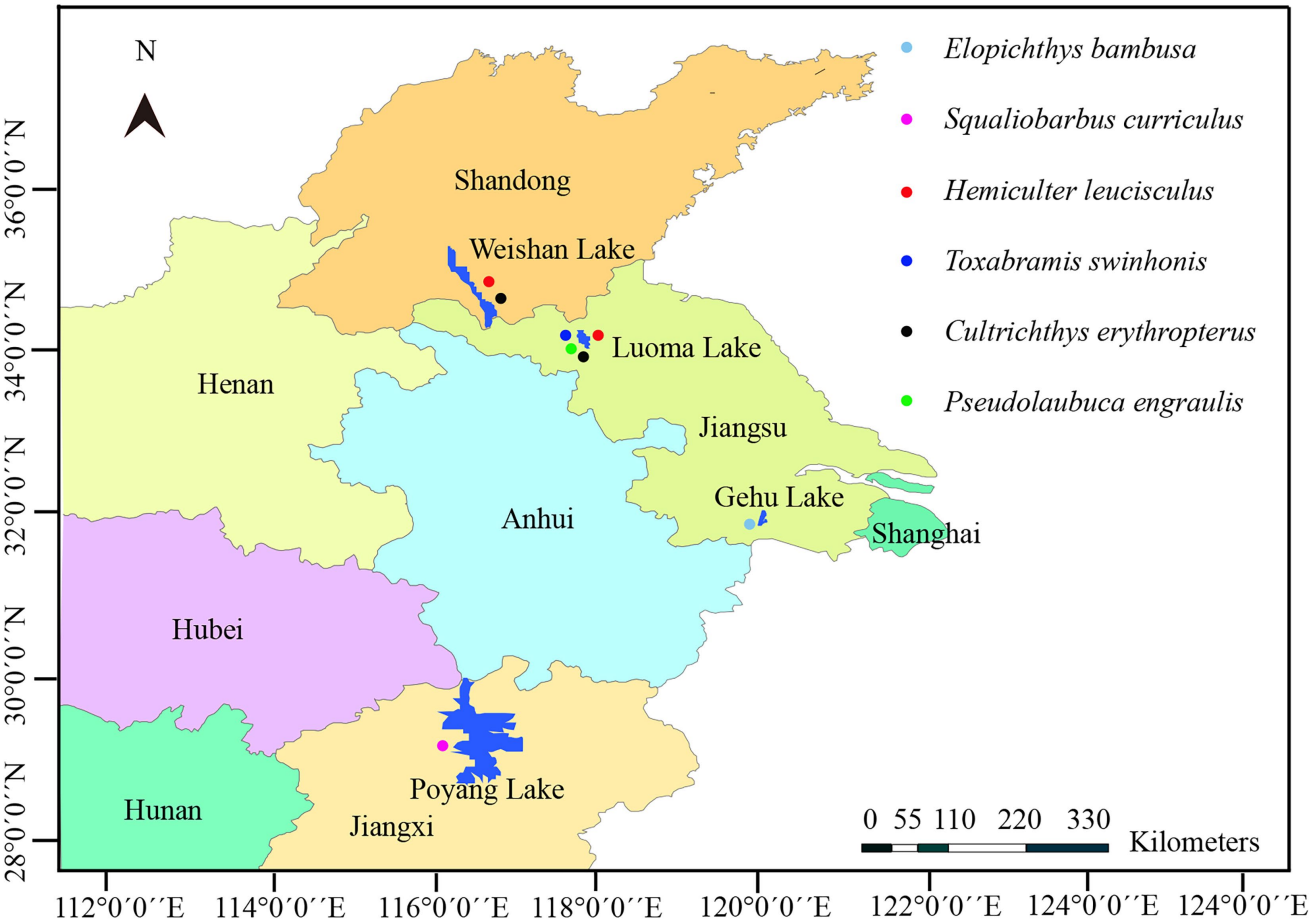
A map of part of Eastern China shows the sampling sites of six species of fish.

Fish specimens of six species were collected from four different Lakes distributed along the middle and lower reaches of the Yangtze River (Figure 1). In detail, 1 specimen of *Squaliobarbus curriculus* was obtained from a fish market in the lakeshore of Lake Poyang located in Northern Jiangxi province, China (29^°^27′3′′N, 116^°^01′32′′E) in July 2018. Six specimens of *Hemiculter leucisculus* and 9 specimens of *Cultrichthys erythropterus* were captured by gill nets from Lake Weishan located in the Southern Shandong province, China (34°36′3.64′′N, 117°20′8.59′′E) in October 2020. One specimen of *Pseudolaubuca engraulis*, 5 specimens of *Hemiculter leucisculus*, 15 specimens of *Cultrichthys erythropterus* and 97 specimens of *Toxabramis swinhonis* were captured by gill nets from Lake Luoma located in Northern Jiangsu province, East China (34^°^4′25.58′′N, 118^°^11′20.38′′E) in October 2020. Two specimens of *Elopichthys bambusa* was collected from Lake Gehu located in Southern Jiangsu province, East China (31^°^36′29.6′′N, 119^°^49′49.76′′E) in May 2022. All fish specimens were immediately transported to the local laboratory in cooled plastic bags for the preliminary parasitological examination. Gross observations were conducted on scales, skin, and visceral organs, including gill, liver, heart, spleen, kidney, intestine, ovary, gallbladder, urinary bladder, and mesentery to find the suspected microsporidian infection for the presence of the whitish cysts which were subjected for the further wet mount preparation. A single cyst isolated from the infected tissues was ruptured with a fine needle on a slide for morphological observation. Spore images were captured using an Olympus BX 53 microscope equipped with an Olympus DP72 digital camera (Olympus, Japan). The cyst-containing tissue samples were preserved in 95% ethanol for further molecular characterization, and in 2.5% glutaraldehyde in 0.1 M sodium cacodylate buffer (pH 7.4) for electron microscopic observation, as well as in 10% neutral buffered formalin for histological examination, respectively. In addition, the previously collected isolate from *Culter alburnus* from Lake Poyang was also used for molecular analyses (Liu et al., 2019).

### 2.2. Histological examination

The tissue samples fixed in 10% neutral-buffered formalin were dehydrated through a series of graded concentration of ethanol, and then embedded in paraffin wax. Tissue sections of 3 μm in width were stained with hematoxylin and eosin (H&E) and examined under Olympus BX 53 microscope (Olympus, Japan).

### 2.3. Transmission electron microscopy observation

Glutaraldehyde-fixed cysts were washed three times for 10 min in sodium cacodylate buffer and then fixed with 1% osmium tetroxide (OsO4) in the same buffer for 1 h. After dehydration through a gradually ascending series of ethanol and propylene oxide, samples were embedded in Spur resin (Reynolds, 1963). Ultrathin sections (70–90 nm) were mounted on an uncoated copper grid and stained with uranyl acetate and lead citrate. Sections of two cysts were examined using a Hitachi HT-7700 transmission electron microscope (TEM).

### 2.4. DNA extraction, polymerase chain reaction, and sequencing

Ethanol-fixed cysts were washed with distilled water two times to remove ethanol remnants. Multiple cysts from a single host were used to extract the genomic DNA. The genomic DNA was extracted using the Qiagen DNeasy Blood & Tissue Kit (Qiagen, Germany) with the assistance of a Fast Prep cell disruptor (FastPrep- 24 5G, MP) for 2 min at 8 m/s following the manufacturer’s instructions. The primer sets used for rDNA and Rpb1 gene amplification were shown in Table 1. Polymerase chain reaction (PCR) was carried out in a 50 μl reaction system, containing PCR buffer, 200 mM dNTP, 2 mM MgCl_2_, 1.25 units Taq polymerase, 20 pmol each primer, and 2 μl DNA template. The partial SSU rDNA was amplified using the primer pair V1f/1492r, and the PCR reaction conditions consisted of an initial denaturation step at 95°C for 4 min, followed by 35 cycles at 95°C for 1 min, 50°C for 30s, 72°C for 2 min, and a final extension at 72°C for 10 min. The 3′ terminal partial SSU rDNA, complete ITS, and the partial LSU rDNA sequence was amplified using the primer pair HG4F/ILSUR, and the PCR cycle comprised of an initial denaturation step at 95°C for 4 min, followed by 35 cycles at 95°C for 30s, 53°C for 30s, 72°C for 2 min, and a final extension at 72°C for 10 min. For some taxa, to which the primer pair HG4F/ILSUR did not work, the primer pair HG4F/HG4R was applied, with thermocycler parameters as follows: an initial denaturation step at 94°C for 2 min, followed by 35 cycles at 94°C for 30 s, 54°C for 30s, 72°C for 1 min, and a final extension at 72°C for 10 min. For amplification of the largest subunit of RNA Polymerase II (Rpb1), the general microsporidian primer pair RPB1-F1/RPB1-R1 was used to obtain preliminary sequence. The preliminary sequence was then used as the template to design specific primers using Primer Premier 5.0. Then, the desired Rpb1 fragement was assembled with three overlapped amplified fragments using the primer pairs AF3/PR1, PF1/RPB1-R1, and PF2/GR1. The PCR amplification was performed under the following conditions: an initial denaturation for 4 min at 94°C, 35 cycles of 30s at 94°C, 30s at 46°C, 1 min at 72°C, and a terminal extension of 10 min at 72°C. All desired PCR products were excised from an agarose gel, purified using a PCR purification kit (CWBIO, Jiangsu, China), and cloned into the PMD-18 T vector system (Takara, Tokyo, Japan). For each gene and isolate, five positive clones were randomly selected for sequencing in both directions with the ABI BigDye Terminator v3.1 Cycle Sequencing Kit and an ABI 3100 Genetic Analyzer.

**Table 1:** Primers used for amplifying and sequencing of microsporidia rDNA and Rpb1 genes.

Gene	Primer	Sequence(5′-3′)	Reference
rRNA	V1F	CACCAGGTTGATTCTGCC	Nilsen (2000)
rRNA	1492R	GGTTACCTTGTTACGACTT	Nilsen (2000)
rRNA	HG4F	GCGGCTTAATTTGACTCAAC	Gatehouse and Malone (1998)
rRNA	HG4R	TCTCCTTGGTCCGTGTTTCAA	Gatehouse and Malone (1998)
rRNA	ILSUR	ACCTGTCTCACGACGGTCTAAAC	Tsai et al. (2002)
Rpb1	AF3	GGWCATTTCGGWCACATIGA	Cheney et al. (2001)
Rpb1	PR1	CAGCCATAGTTTGATACG	Herein
Rpb1	PF1	TGCCTCAGTCGTATCAA	Herein
Rpb1	PRB1F1	CGGACTTYGAYGGNGAYGARATGA	Hirt et al. (1999)
Rpb1	PRB1R1	CCCGCKNCCNCCCATNGCRTGRAA	Hirt et al. (1999)
Rpb1	PF2	TAAGAATGACTATGGCTGTG	Herein
Rpb1	GR1	TGRAAMGTRTTIAGIGTCATYTG	Cheney et al. (2001)

### 2.5. Phylogenetic analysis

The all obtained sequences fragments were assembled by BioEdit (Hall, 1999) to produce consensus sequences which were applied to determine their genetically identical species by a BLASTn search. To explore the phylogenetic relationships of these sequences, fifteen concatenated SSU-ITS-LSU sequences were retrieved from the GenBank database. The gene sequences were aligned with Clustal X by the default setting (Thompson et al., 1997). The obtained alignment was corrected manually using the alignment editor function involved in MEGA 6.0 (Tamura et al., 2013). The gaps which could not be aligned were excluded from the alignments. *Gurleya daphniae* (AF439320) was used as an outgroup. Pairwise genetic distances/similarities of these species based on SSU, ITS, and LSU were calculated using the Kimura-2 parameter model distance matrix for transitions and transversions. Phylogenetic analyses were conducted using the maximum likelihood (ML) method in PhyML 3.0 and Bayesian inference (BI) in MrBayes 3.2.4, respectively. The optimal evolutionary model was determined to be GTR + I + G by ModelTest 3.7 using the Akaike information criteria. Two independent runs were conducted with four chains for one million generations for BI. Phylogenetic trees were sampled every 100 generations. The first 25% of the samples were discarded from the cold chain (burninfrac = 0.25). Bootstrap confidence values were calculated with 100 repetitions for ML. The tree was initially examined in Figtree v1.4.4^1^, edited, and annotated in Adobe Illustrator (Adobe System, San Jose, CA, United States).

### 2.6. Genetic analysis

Changes in nucleotide diversity of rDNA and Rpb1 gene were calculated as the average heterozygosity per site (π) and the average number of nucleotide differences per site (θ_w_) by using DnaSP v5.10 (Librado and Rozas, 2009). The neutrality tests Tajima’s *D* (Tajima, 1989) and Fu’s *F*s (Fu, 1997) were computed using DnaSP v5.10 and Arlequin 3.5 (Excoffier and Lischer, 2010), respectively. Fixation indices (*F*st) were calculated to evaluate population genetic differentiation using Arlequin 3.5. Molecular variance analysis (AMOVA) (Excoffier et al., 1992) was conducted in Arlequin 3.5. Haplotype network was calculated using the Median Joining (MJ) method in Network 10^2^.

The genetic recombination was analyzed with RDP4 (Martin et al., 2015) and SimPlot (Lole et al., 1999). First, the recombination analysis was implemented in the RDP4 by using the available 7 methods (RDP, 3Seq, GENECONV, BoostScan, MaxChi, Chimaera, and SiScan) with default parameters. Detected events supported by at least three of the seven methods were considered as recombination. The recombination events detected by RDP4 were further analyzed in SimPlot. The final recombination events were determined based on the combined results from SimPlot and RDP4 analysis.

## 3. Results

### 3.1. Macroscopical, light microscopical observations, and gross pathology

**Figure 2:**
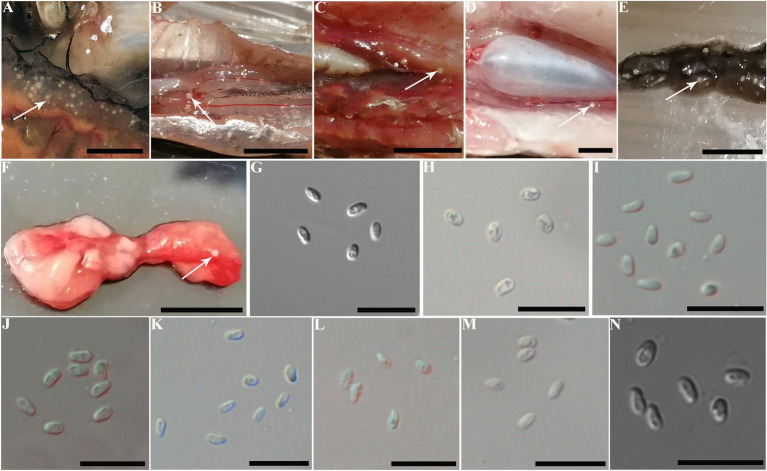
Macroscopical observation and light microscopy of *Pseudokabatana alburnus* isolated from the ovary of different fishes from different geographical locations. **(A–F)** A gallery of cysts (arrow) of *P. alburnus* located in the ovary of different fish, demonstrating the similar morphology of cysts. **(A)** *Squaliobarbus curriculus.* **(B)** *Hemiculter leucisculus.* **(C)** *Pseudolaubuca engraulis.* **(D)** *Cultrichthys erythropterus.* **(E)** *Toxabramis swinhonis.* **(F)** *Elopichthys bambusa.* Scale bar = 10 mm. **(G–N)** A gallery of *P. alburnus* spores from various fish species, demonstrating the similar spore morphology. **(G)** Spores isolated from *S. curriculus* from Poyang Lake. **(H)** Spores isolated from *H. leucisculus* from Luoma Lake. **(I)** Spores isolated from *P. engraulis* from Luoma Lake. **(J)** Spores isolated from *C. erythropterus* from Luoma Lake. **(K)** Spores isolated from *T. swinhonis* from Luoma Lake. **(L)** Spores isolated from *H. leucisculus* from Weishan Lake. **(M)** Spores isolated from *C. erythropterus* from Weishan Lake. **(N)** Spores isolated from *E. bambusa* from Gehu Lake. Scale bar = 10 μm.

**Figure 3:**
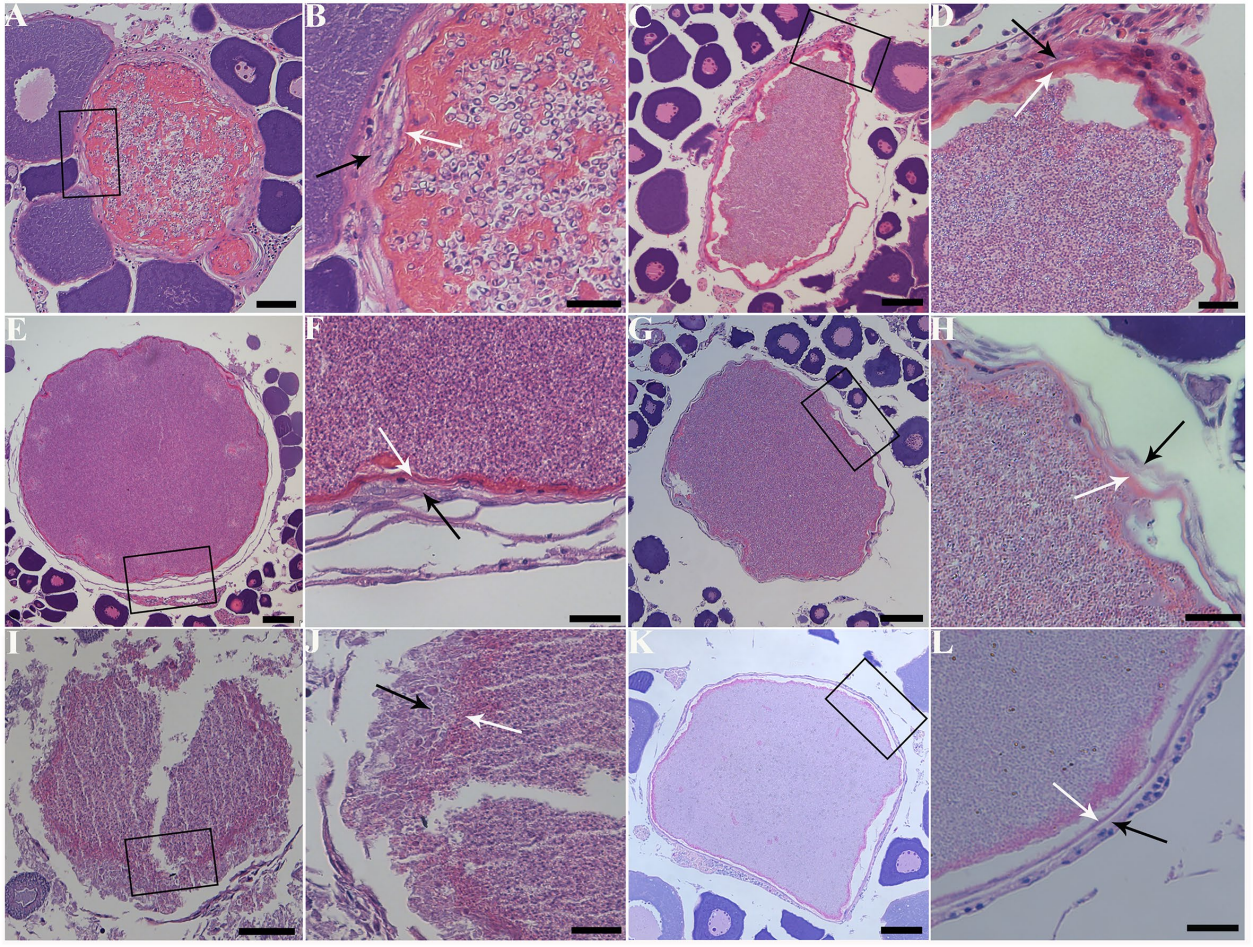
Histopathology of the ovary of six fish species with infection of *Pseudokabatana alburnus*. **(A,C,E,G,I,K)** A gallery of large parasite-filled cysts isolated from different fish species, demonstrating cysts developing in the connective tissue between the oocytes. **(B,D,F,H,J,L)** A gallery of magnified images of different pictures, showing the cyst wall (white arrow) surrounded with connective tissue (black arrow). **(A)** *Cultrichthys erythropterus*. Scale bar = 50 μm. **(B)** The boxed area in **(A)**. Scale bar = 20 μm. **(C)** *Pseudolaubuca engraulis*. Scale bar = 50 μm. **(D)** The boxed area in **(C)**. Scale bar = 20 μm. **(E)** *Hemiculter leucisculus*. Scale bar = 50 μm. **(F)** The boxed area in **(E)**. Scale bar = 20 μm. **(G)** *Toxabramis swinhonis*. Scale bar = 50 μm. **(H)** The boxed area in **(G)**. Scale bar = 20 μm. **(I)** *Squaliobarbus curriculus*. Scale bar = 50 μm. **(J)** The boxed area in **(I)**. Scale bar = 20 μm. **(K)** *Elopichthys bambusa*. Scale bar = 50 μm. **(L)** The boxed area in **(K)**. Scale bar = 20 μm.

The infected sites presented as macroscopic whitish nodules of variable sizes and shapes, which were solely found in the ovary of examined fish samples. The details of cyst prevalence were documented as follows: 1 out of 1 *Squaliobarbus curriculus* (Figure 2A) sampled from Lake Poyang; 1 out of 5 *Hemiculter leucisculus* (Figure 2B), 1 out of 1 *Pseudolaubuca engraulis* (Figure 2C), 2 out of 15 *Cultrichthys erythropterus* (Figure 2D), 15 out of 97 *Toxabramis swinhonis* (Figure 2E) sampled from Lake Luoma; 2 of 9 *C. erythropterus*, 1 out of 6 *H. leucisculus* sampled from Lake Weishan; 1 out of 2 *Elopichthys bambusa* (Figure 2F) sampled from Lake Gehu. Cysts in different fishes differed in size (Figures 2A–F). The largest cysts from *C. erythropterus* collected from Lake Luoma were spherical to oval, measuring 1.34 (0.70–1.85) mm long, and 1.11 (0.71–1.41) mm wide (N = 10). The smallest cysts from *S. curriculus* collected from Lake Poyang were spherical to oval, measuring 0.45 (0.34–0.60) mm long and 0.40 (0.30–0.56) mm wide (*N* = 10) (Table 2). Spores isolated from different fish have different sizes (Figures 2G–N). The largest spores isolated from *T. swinhonis* collected from Lake Luoma measured 3.54 ± 0.17 (3.20–3.85) μm in length and 1.90 ± 0.11 (1.60–2.05) μm in width (*N* = 40). The smallest spores isolated from *H. leucisculus* collected from Lake Weishan were pyriform, and measured 2.96 ± 0.21 (2.65–3.27) μm in length and 1.66 ± 0.09 (1.46–1.82) μm in width (*N* = 40) (Table 2). Histological analysis showed that all cysts from these fish developed in the connective tissue of ovary parenchyma between oocytes (Figure 3). The wall of cysts is very thin, and no obvious inflammatory response was observed in the infected tissues, regardless of their host species and sources.

**Table 2:** Morphological comparison of different *Pseudokabatana alburnus* isolates.

Host	Infected site	Prevalence	Location	Cysts size (mm)	Spores size (μm)	Spore wall thickness (nm)	References
*Pseudolaubuca engraulis*	Ovary	100%	Lake Luoma	1.0 × 1.0	3.5 × 1.8	68–84	Herein
*Cultrichthys erythropterus*	Ovary	13.3%	Lake Luoma	1.34 × 1.11	3.4 × 1.9	56–75	Herein
*Toxabramis swinhonis*	Ovary	6.5%	Lake Luoma	0.84 × 0.84	3.5 × 1.9	64–80	Herein
*Hemiculter leucisculus*	Ovary	20%	Lake Luoma	0.89 × 0.69	3.3× 2.3	-	Herein
*Hemiculter leucisculus*	Ovary	16.7%	Lake Weishan	-	3.0 × 1.7	80–97	Herein
*Cultrichthys erythropterus*	Ovary	22.2%	Lake Weishan	-	3.3 × 1.8	-	Herein
*Squaliobarbus curriculus*	Ovary	100%	Lake Poyang	0.45 × 0.40	3.5 × 1.8	-	Herein
*Elopichthys bambusa*	Ovary	50%	Lake Gehu	0.86 × 0.86	3.3 × 2.3	82–113	Herein
*Culter alburnus*	Liver	50%	Lake Poyang	1.20 × 1.20	2.3 × 1.3	120–165	(Liu et al., 2019)

### 3.2. Transmission electron microscopy

**Figure 4:**
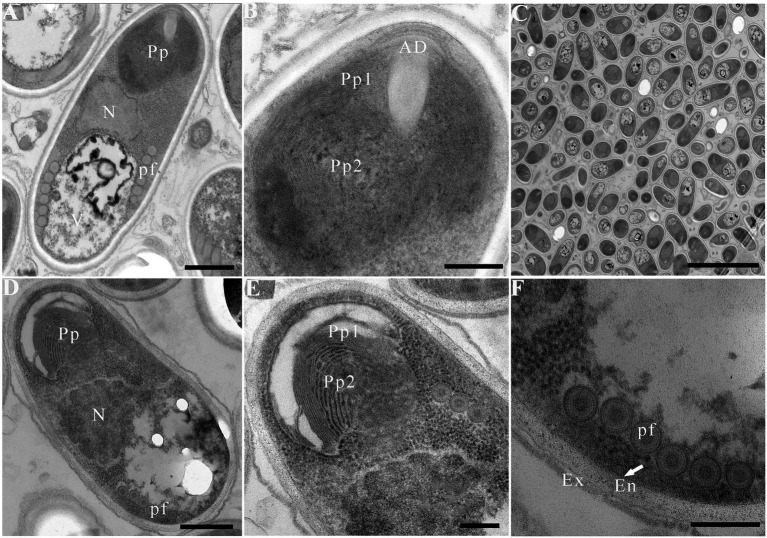
Electron microscopy of *Pseudokabatana alburnus* isolated from different fish. **(A–C)** Mature spore isolated from *Toxabramis swinhonis*, **(D–F)** Mature spore isolated from *Hemiculter leucisculus.* **(A)** A mature spore of *P. alburnus* isolated from *T. swinhonis* with typical microsporidian features of internal structure, including a mushroom-shaped anchoring disk, a bipartite polaroplast (Pp), a large nucleus (N), a large vacuole (V), the isofilar polar filaments (pf) and a trilaminar spore wall. Scale bar = 500 nm. **(B)** Magnification of the bipartite polaroplast showing narrow lamellae (Pp1) and wide lamellae (Pp2), a mushroom-shaped anchoring disc (AD) locating in the apex of spore. Scale bar = 200 nm. **(C)** Mature spores occur in direct contact with the host cell cytoplasm. Scale bar = 5 μm. **(D)** A mature spore of *P. alburnus* isolated from *H. leucisculus* with bipartite polaroplast (Pp) and the isofilar polar filaments (pf). Scale bar = 500 nm. **(E)** Magnification of bipartite polaroplast (Pp1, Pp2). Scale bar = 200 nm. **(F)** Magnification of trilaminar spores wall showing an electron-dense exospore (Ex), an electron-translucent endospore (En) and a plasma membrane (arrow). Scale bar = 200 nm.

**Figure 5:**
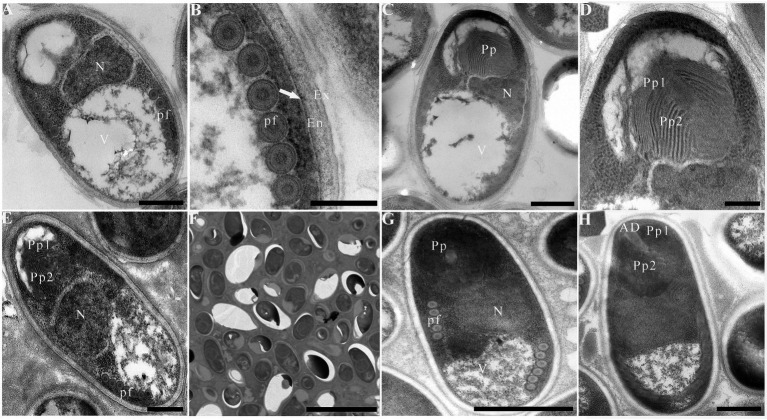
Electron microscopy of *Pseudokabatana alburnus* isolated from different fish. **(A)** A mature spore of *P. alburnus* isolated from *Cultrichthys erythropterus* showing six coils of polar filament (pf), a vacuole (V) and a large nucleus (N). Scale bar = 500 nm. **(B)** Magnification of trilaminar spores wall showing an electron-dense exospore (Ex), an electron-translucent endospore (En) and a plasma membrane (arrow). Scale bar = 200 nm. **(C)** A mature spore of *P. alburnus* isolated from *C. erythropterus* with bipartite polaroplast, a vacuole and a large nucleus. Scale bar = 500 nm. **(D)** Magnification of bipartite polaroplast (Pp1, Pp2). Scale bar = 200 nm. **(E)** A mature spore of *P. alburnus* isolated from *Pseudolaubuca engraulis* with a bipartite polaroplast (Pp1, Pp2), a large nucleus (N) and the isofilar polar filaments (pf). Scale bar = 500 nm. **(F)** Mature spores occur in direct contact with the host cell cytoplasm. Scale bar = 5 μm. **(G)** A mature spore of *P. alburnus* isolated from *Elopichthys bambusa* showing 5–6 coils of polar filament (pf), a vacuole (V) and a large nucleus (N). Scale bar = 500 nm. **(H)** A mature spore of *P. alburnus* isolated from *E. bambusa* showing bipartite polaroplast (Pp1, Pp2) and a mushroom-shaped anchoring disk (AD). Scale bar = 500 nm.

**Figure 6:**
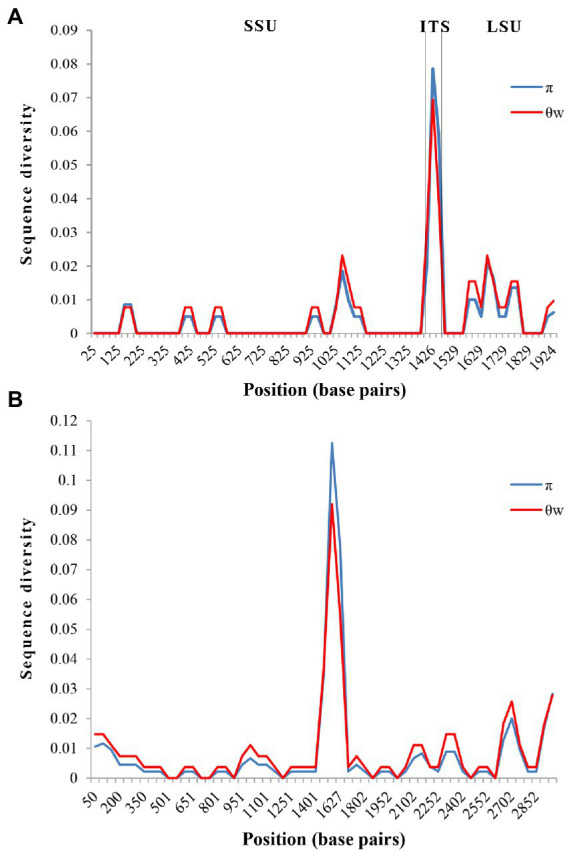
Sliding window analysis of nucleotide diversity in the rDNA and Rpb1 of *Pseudokabatana alburnus.* Nucleotide diversity was calculated as the average heterozygosity per site (π) and the average number of nucleotide differences per site (*θ*_w_). **(A)** Sliding window analysis of nucleotide diversity in the rDNA of *P. alburnus.* A sliding window of 50 base pairs was used, with an increment of 25 base pairs. **(B)** Sliding window analysis of nucleotide diversity in the Rpb1 of *P. alburnus.* A sliding window of 100 base pairs was used, with an increment of 50 base pairs.

Due to insufficient samples, only suspected microsporidian cysts isolated from *T. swinhonis* from Lake Luoma, *H. leucisculus* from Lake Weishan, *C. erythropterus* from Lake Luoma, *P. engraulis* from Lake Luoma, and *E. bambusa* from Lake Gehu were subjected to the TEM investigation. For being almost the most advanced development stage, ultrastructural characteristics of mature spores from the above 5 specimens were solely presented here which are highly similar, but also of slight difference in spore size (Table 2). Mature spores isolated from *T. swinhonis* from Lake Luoma are pyriform, and are in direct contact with the host cell cytoplasm (Figures 4A,C). Uninucleate spores possess 5–6 isofilar polar filaments (Figure 4A), a large posterior vacuole (Figure 4A), a mushroom-shaped anchoring disc, and bipartite polaroplast (closely packed lamellae and wider lamellae) (Figure 4B). The spore wall exhibits typical three layers, including a 20–27 nm thick electron-dense exospore, a 36–45 nm thick electron-lucent endospore, and an 8 nm thick plasma membrane (Figure 4B). Mature spores isolated from *H. leucisculus* from Lake Weishan are monokaryotic and pyriform (Figure 4D). The spores have bipartite polaroplast (tight lamellae and loose lamellae) (Figure 4E), and 5–6 isofilar polar filaments coils in one row (Figure 4F). Their spore wall consists of a 20–30 nm thick electron-dense exospore, a 55–60 nm thick electron-lucent endospore, and a 5–7 nm thick plasma membrane (Figure 4F). Mature spores isolated from *C. erythropterus* from Lake Luoma ultrastructurally resemble with those from *H. leucisculus*. Uninucleate spores possess a large posterior vacuole (Figures 5A,C), 5–6 polar filament coils in a single row (Figure 5B), and bipartite polaroplast (narrow lamellae and wide lamellae) (Figures 5C,D). The spore wall consists of an 18–25 nm thick electron-dense exospore, a 33–43 nm thick electron-lucent endospore, and a 5–7 nm thick plasma membrane (Figure 5B). Mature spores isolated from *P. engraulis* from Lake Luoma are monokaryotic, pyriform, and are in direct contact with the host cell cytoplasm (Figures 5E,F). The spores have bipartite polaroplast (tight lamellae and loose lamellae) (Figure 5E), and 5–6 polar filaments coils in one row (Figure 5E). The spore wall is trilaminar, including a 19–21 nm thick electron-dense exospore, a 43–55 nm thick electron-lucent endospore, and a 6–8 nm thick plasma membrane. Mature spores isolated from *E. bambusa* from Lake Gehu are pyriform. Uninucleate spores possess a large posterior vacuole (Figure 5G), 5–6 polar filament coils (Figure 5G), a mushroom-shaped anchoring disc, and bipartite polaroplast (narrow lamellae and wide lamellae) (Figure 5H).

### 3.3. Molecular characterization

**Figure 7:**
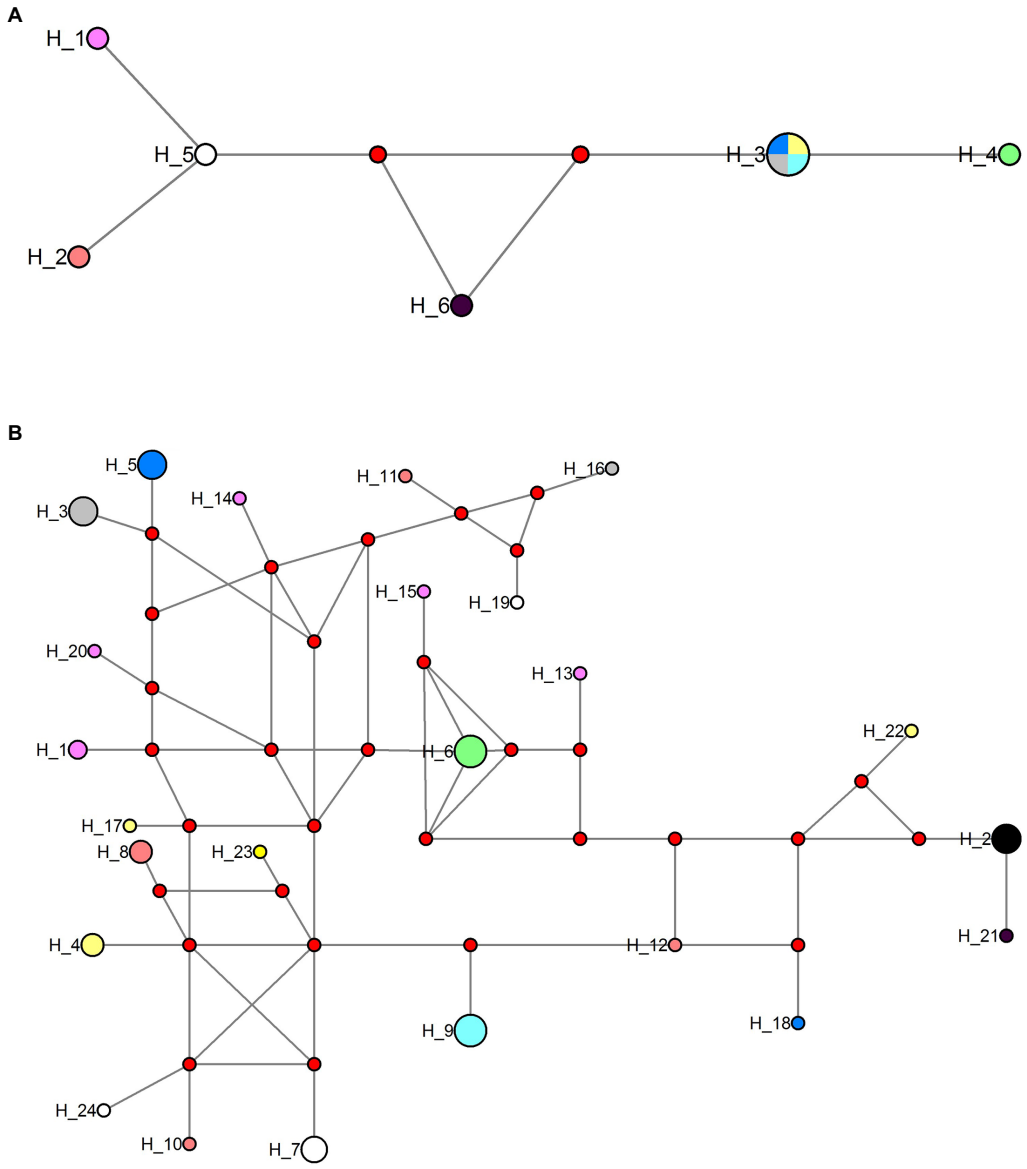
Haplotype network of *Pseudokabatana alburnus* from different hosts and geographical origin using the ITS and Rpb1 sequences data set. **(A)** Haplotype network of *P. alburnus* isolates using ITS sequence data set. **(B)** Haplotype network of *P. alburnus* isolates using Rpb1 sequences data set. Haplotypes are depicted by circles, the width being proportional to their frequencies. Red circle indicates a single connection step between the Rpb1 sequences. Color codes are as follows; light red: haplotypes obtained from *Squaliobarbus curriculus* (Lake Poyang); pink: haplotypes obtained from *Hemiculter leucisculus* (Lake Luoma); black: haplotypes obtained from *Pseudolaubuca engraulis* (Lake Luoma); gray: haplotypes obtained from *Cultrichthys erythropterus* (Lake Luoma); yellow: haplotypes obtained from *Toxabramis swinhonis* (Lake Luoma); dark blue: haplotypes obtained from *Culter alburnus* (Lake Poyang); green: haplotypes obtained from *H. leucisculus* (Lake Weishan); white: haplotypes obtained from *C. erythropterus* (Lake Weishan); wathet: haplotypes obtained from *Elopichthys bambusa* (Lake Gehu).

**Figure 8:**
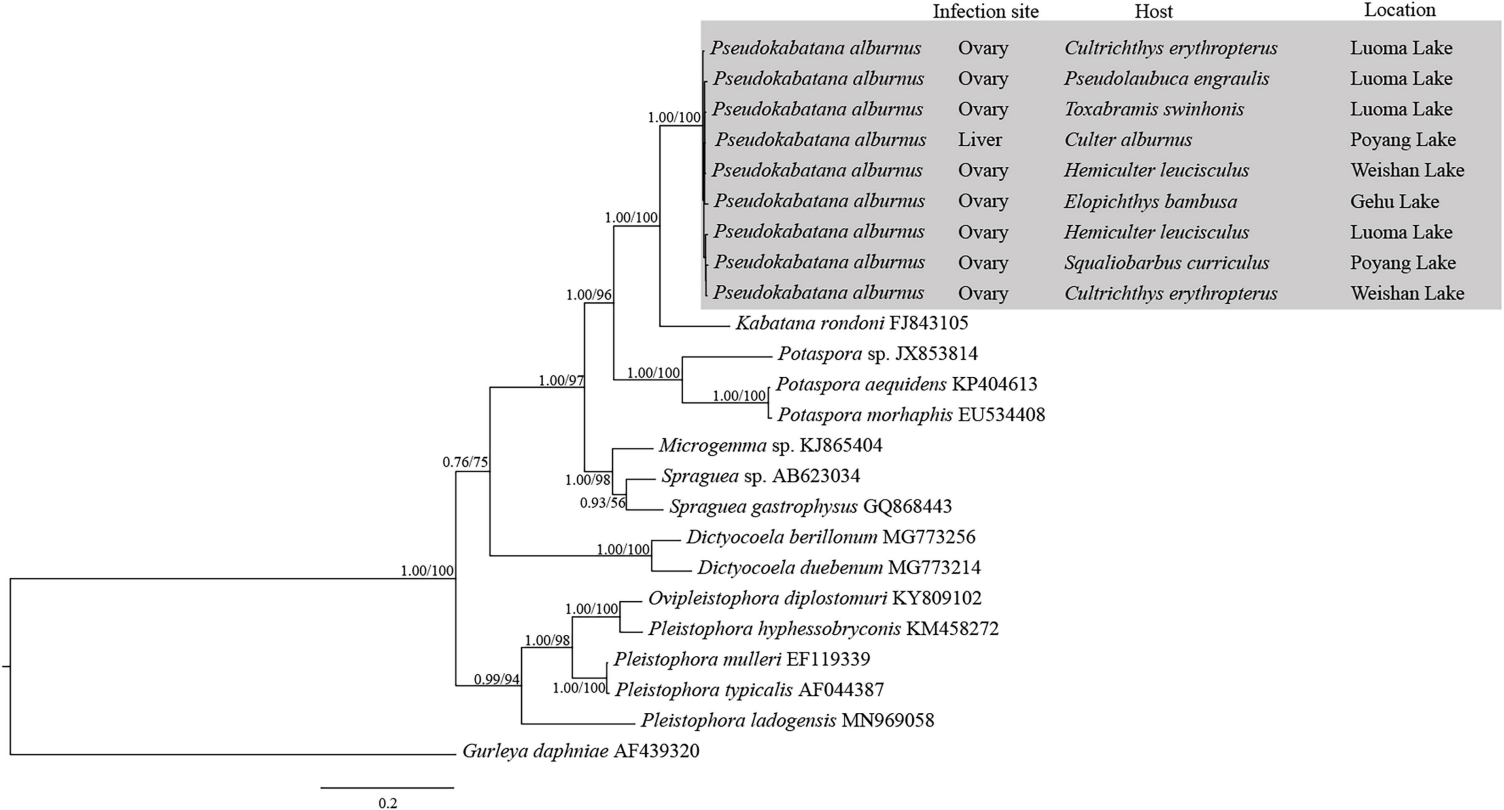
The rDNA unit (partial SSU + ITS + partial LSU)-based phylogenetic relationships between *Pseudokabatana alburnus* and the other aligned microsporidian species by Bayesian Inference (BI) method. The species names are followed by GenBank accession number. BI posterior probabilities were shown firstly, followed by ML support values on branch nodes. The infection site and host, as well as the geographical location of *P. alburnus* were presented.

The obtained SSU rDNA sequences of all microsporidians isolated here were 1,383 bp in length and the consensus sequences were deposited in GenBank under accession numbers OM423155-OM423161 and OP942336. Sequence comparison showed that they are almost identical, with sequence identity of 99.68–99.92%, and BLASTn search showed that they are highly similar to that of *P. alburnus* (MF974572, with sequence similarity of 99.84–99.92%) recently identified from the liver of *Culter alburnus* in Lake Poyang, followed by that of *Kabatana rondoni* (FJ843105, with sequence similarity of 90.02–90.21%), *Inodosporus octosporus* (MH911929, with sequence similarity of 88.86–89.06%), and *K. takedai* (AF356222, with sequence similarity of 87.67–87.96%). Therefore, based on the morphological and high SSU rDNA sequences similarity, the microsporidian from the ovary of all sampled 6 East Asian minnows along the middle and lower reaches of the Yangtze River are conspecific to *P. alburnus*. The pairwise distances/sequence similarities between the obtained SSU rDNA sequences of all *P. alburnus* isolates and 3 highly genetically related *Kabatana* species ranged from 0.0008/99.92% (between the *P. alburnus* isolated from *C. alburnus* from Lake Poyang and the *P. alburnus* isolated from *P. engraulis* from Lake Luoma) to 0.1340/86.60% (between *K. rondoni* FJ843105 and *K. takedai* AF356222) (Table 3). Given low resolution of SSU rDNA to discern the possible interspecific variation of *P. alburnus*, their full ITS and partial LSU rDNA sequences which were previously proven to cover more variable sites were amplified and assembled, and their concatenated partial SSU, complete ITS and partial LSU were also deposited in GenBank with accession numbers OM423163-OM423170 and OP948229. Sequence comparisons showed that there was significantly different genetic variation among the ITS sequences of all isolates of *P. alburnus*, with 79.6–100% sequence similarity (Table 4), however, their LSU rDNA sequences are highly similar, with 98.96–99.79% sequence similarity (Table 5). Genetic diversity analysis showed that the π and *θ*_w_ value in SSU, ITS and LSU loci was 0.00314 ± 0.00038, 0.07341 ± 0.01561 and 0.00594 ± 0.00096, respectively, and was 0.00399 ± 0.00103, 0.07008 ± 0.02478 and 0.00757 ± 0.00239, respectively (Table 6). Sliding window analysis of rDNA locus of *P. alburnus* isolates further demonstrated that the ITS region has higher nucleotide diversity than that of SSU and LSU region. The value of π and *θ*_w_ followed similar variation pattern across all sliding windows for the rDNA locus (Figure 6A). Haplotype networks indicate that ITS haplotypes isolated from same location did not clearly cluster with the host species and resource (Figure 7A). Bayesian and maximum likelihood analyses of the aligned concatenated partial SSU, ITS and partial LSU of 24 microsporidian species produced phylogenetic trees of highly similar topologies, although with different support values at some branch nodes. The results showed that all *P. alburnus* isolates clustered to an independent branch, but infection sites, host species, and host resource did not provide distinct phylogenetic signal (Figure 8).

**Table 3:** Pairwise nucleotide sequence identity (upper right) values and evolutionary distances (left bottom) among *Pseudokabatana alburnus* isolates and 3 other microsporidian species with high sequence similarity by Kimura-2 Parameter analysis based on SSU rDNA sequences.

Species (Host, Location or GenBank accession number)	1	2	3	4	5	6	7	8	9	10	11	12
1. *Pseudokabatana alburnus* (*Toxabramis swinhonis*, Lake Luoma)	–	99.84	99.76	99.76	99.68	99.68	99.68	99.68	99.68	90.21	89.06	87.96
2. *P. alburnus* (*Culter alburnus*, Lake Poyang)	0.0016	–	99.92	99.92	99.84	99.84	99.84	99.84	99.84	90.21	89.06	87.87
3. *P. alburnus* (*Pseudolaubuca engraulis*, Lake Luoma)	0.0024	0.0008	–	99.84	99.76	99.76	99.76	99.76	99.76	90.11	88.96	87.77
4. *P. alburnus* (*Hemiculter leucisculus*, Lake Weishan)	0.0024	0.0008	0.0016	–	99.76	99.76	99.76	99.76	99.76	90.11	88.96	87.77
5. *P. alburnus* (*Cultrichthys erythropteru*, Lake Luoma)	0.0032	0.0016	0.0024	0.0024	–	99.84	99.68	99.68	99.84	90.20	88.86	87.86
6. *P. alburnus* (*Hemiculter leucisculus*, Lake Luoma)	0.0032	0.0016	0.0024	0.0024	0.0016	–	99.68	99.68	99.84	90.20	88.86	87.86
7. *P. alburnus* (*Squaliobarbus curriculus*, Lake Poyang)	0.0032	0.0016	0.0024	0.0024	0.0032	0.0032	-	99.68	99.68	90.02	88.86	87.67
8. *P. alburnus* (*Cultrichthys erythropterus*, Lake Weishan)	0.0032	0.0016	0.0024	0.0024	0.0032	0.0032	0.0032	-	99.68	90.21	88.96	87.87
9. *P. alburnus* (*Elopichthys bambusa*, Lake Gehu)	0.0032	0.0016	0.0024	0.0024	0.0016	0.0016	0.0032	0.0032	-	90.21	88.87	87.87
10. *Kabatana rondoni* (FJ843105)	0.0979	0.0979	0.0989	0.0989	0.0980	0.0980	0.0998	0.0979	0.0979	-	87.33	86.60
11. *Inodosporus octosporus* (MH911629)	0.1094	0.1094	0.1104	0.1104	0.1114	0.1114	0.1114	0.1104	0.1113	0.1267	–	93.38
12. *K. takedai* (AF356222)	0.1204	0.1213	0.1223	0.1223	0.1214	0.1214	0.1233	0.1213	0.1213	0.1340	0.0662	–

**Table 4:** Pairwise nucleotide sequence identity (upper right) values and evolutionary distances (left bottom) among *P. alburnus* isolates isolated from different hosts and locations by Kimura-2 Parameter analysis based on ITS rDNA sequences.

Host (Location)	1	2	3	4	5	6	7	8	9
1. *Toxabramis swinhonis* (Lake Luoma)	–	100.00	100.00	97.84	97.83	95.41	83.39	88.52	82.54
2. *Culter alburnus* (Lake Poyang)	0.0000	–	100.00	97.84	97.83	95.41	83.39	88.52	82.54
3. *Elopichthys bambusa* (Lake Gehu)	0.0000	0.0000	–	97.79	97.78	95.31	82.99	88.25	82.09
4. *Cultrichthys erythropterus* (Lake Luoma)	0.0216	0.0216	0.0221	–	95.61	95.41	86.01	90.95	85.27
5. *Hemiculter leucisculus* (Lake Weishan)	0.0217	0.0217	0.0222	0.0439	–	92.97	80.69	85.96	79.60
6. *Pseudolaubuca engraulis* (Lake Luoma)	0.0459	0.0459	0.0469	0.0459	0.0703	-	87.83	92.97	87.34
7. *Hemiculter leucisculus* (Lake Luoma)	0.1661	0.1661	0.1701	0.1399	0.1931	0.1217	–	95.60	93.02
8. *Cultrichthys erythropterus* (Lake Weishan)	0.1148	0.1148	0.1175	0.0905	0.1404	0.0703	0.0440	–	95.41
9. *Squaliobarbus curriculus* (Lake Poyang)	0.1746	0.1746	0.1791	0.1473	0.2040	0.1266	0.0698	0.0459	–

**Table 5:** Pairwise nucleotide sequence identity (upper right) values and evolutionary distances (left bottom) among *P. alburnus* isolates isolated from different hosts and locations by Kimura-2 Parameter analysis based on LSU rDNA sequences.

Host (Location)	1	2	3	4	5	6	7	8	9
1. *Toxabramis swinhonis* (Lake Luoma)	–	99.38	99.38	99.17	99.17	99.17	98.96	98.96	98.96
2. *Cultrichthys erythropterus* (Lake Luoma)	0.0062	–	99.59	99.38	99.38	99.79	99.17	99.59	99.59
3. *Hemiculter leucisculus* (Lake Luoma)	0.0062	0.0041	–	99.79	99.79	99.79	99.59	99.59	99.59
4. *Pseudolaubuca engraulis* (Lake Luoma)	0.0083	0.0062	0.0021	–	99.59	99.59	99.38	99.38	99.38
5. *Hemiculter leucisculus* (Lake Weishan)	0.0083	0.0062	0.0021	0.0041	–	99.59	99.38	99.38	99.38
*6. Squaliobarbus curriculus* (Lake Poyang)	0.0083	0.0021	0.0021	0.0041	0.0041	–	99.38	99.79	99.79
7. *Elopichthys bambusa* (Lake Gehu)	0.0104	0.0083	0.0041	0.0062	0.0062	0.0062	-	99.17	99.59
8. *Culter alburnus* (Lake Poyang)	0.0104	0.0041	0.0041	0.0062	0.0062	0.0021	0.0083	-	99.59
9. *Cultrichthys erythropterus* (Lake Weishan)	0.0104	0.0041	0.0041	0.0062	0.0062	0.0021	0.0041	0.0041	-

**Table 6:** Genetic diversity, Tajima’s *D*, and Fu’s *F*s in four gene regions of *Pseudokabatana alburnus*.

Gene	*N*	π ± SD	θ_w_ ± SD	Tajima’s D	Fu’s *F*s
SSU	9	0.00314 ± 0.00038	0.00399 ± 0.00103	−1.03668	−5.40634
ITS	9	0.07341 ± 0.01561	0.07008 ± 0.02478	−0.31702	−1.09718
LSU	9	0.00594 ± 0.00096	0.00757 ± 0.00239	−1.00013	−7.08967
RPB1	24	0.01687 ± 0.00078	0.01940 ± 0.00223	−1.23541	−11.30516

Nucleotide diversity is calculated as the average heterozygosity per site (*π*) and the average number of nucleotide differences per site (*θ*_w_). N, numbers of sequences.

**Figure 9:**
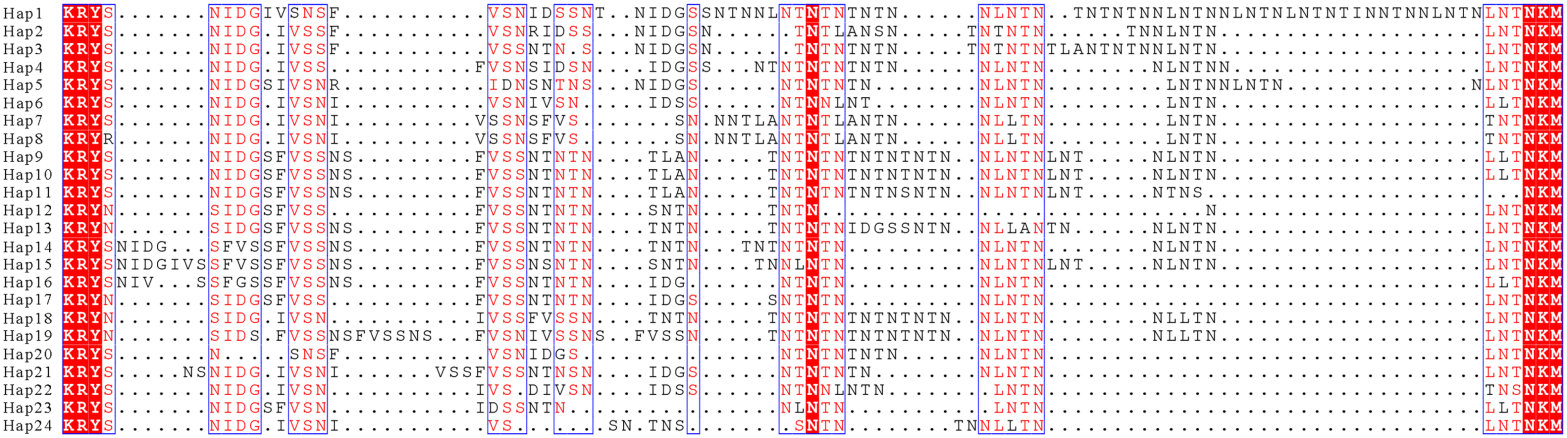
Alignment of the representative Rpb1 protein sequences of 24 haplotypes obtained from nine *Pseudokabatana alburnus* isolates. Dots depict gaps. The red outlined highlighted the identical amino acid. Mostly conserved or similar amino acid are shown in red with the blue boxes.

The Rpb1 gene sequences of some *P. alburnus* isolates were successfully amplified to further explore their possible genetic variation which were deposited in GenBank under accession numbers ON049422–ON049429, ON087855–ON087869, and OP150918, but failed for other isolates. In detail, five Rpb1 sequences were obtained from *H. leucisculus* individual from Lake Luoma. Four Rpb1 sequences were obtained from *S. curriculus* individual from Lake Poyang and of *T. swinhonis* individual from Lake Luoma, respectively. Three Rpb1 sequences were obtained from *C. erythropterus* individual from Lake Weishan. Two Rpb1 sequences were obtained from *P. engraulis* individual from Lake Luoma, of *C. erythropterus* individual from Lake Luoma and of *C. alburnus* individual from Lake Poyang, respectively. Totally, 24 *P. alburnus* haplotypes referring from Rpb1 sequence variation were detected (Figure 9 and Supplementary Figure S1). Genetic diversity analysis showed that the π and *θ*_w_ of the Rpb1 gene were 0.01687 ± 0.00078 and 0.01940 ± 0.00223, respectively (Table 6). Sequence diversity per site based on the sliding window analyses indicated that different regions of Rpb1 gene presented different patterns of variation, and the region of between 1,477–1737 positions had higher nucleotide diversity than the other regions. The value of π and *θ*_w_ followed similar patterns of variation across all sliding windows for Rpb1 locus, which resembled rDNA gene (Figures 6A,B). Haplotype networks also indicated that Rpb1 haplotype distributions resembled ITS haplotype distributions and did not cluster by hosts and geographical origin (Figure 7B).

AMOVA analysis based on RPB1 sequences demonstrated that most of the molecular variance came from the within population (Table 7). The total variability observed among isolates and among host within isolates were −2.44 and −4.70, respectively, whereas 107.14% of the variation were found within populations. The fixation index *F*st based on RPB1 sequences were negative values among isolates, suggesting that there was no genetic differentiation between *P. alburnus* populations (Table 8).

**Table 7:** Analysis of molecular variance (AMOVA) of *Pseudokabatana alburnus* based on Rpb1 gene.

Source of variation	d.f.	SS	VC	%var	*P*
Among isolates	3	19.429	−0.18863 Va	−2.44	ns
Among host within isolates	5	36.554	−0.36338 Vb	−4.70	ns
Within isolates	15	124.267	8.28444 Vc	107.14	ns
Total	23	180.250	7.73243		

d.f., degrees of freedom; SS, sum of squares; VC, variance components; % var., percentage of variation; P, probability of a random variance component value ≤ observed value, ns, non-significant.

**Table 8:** Pairwise genetic differentiation Fst among *Pseudokabatana alburnus* isolates based on Rpb1 gene.

	PY	LM	WS
PY	–	–	–
LM	−0.02717	–	–
WS	−0.08025	−0.04325	–

PY, Lake Poyang; LM, Lake Luoma; WS, Lake Weishan.

The Tajima’s *D* and Fu’s *F*s neutrality test of SSU, ITS, LSU and RPB1 showed negative values of not reaching a significant level (Table 6). The Tajima’s *D* values was −1.03668, −0.31702, −1.00013, and − 1.23541 in SSU, ITS, LSU and RPB1 genes, respectively. The Fu’s *F*s values was −5.40634, −1.09718, −7.08967, and −11.30516 in four loci, respectively.

### 3.4. Recombination

**Figure 10:**
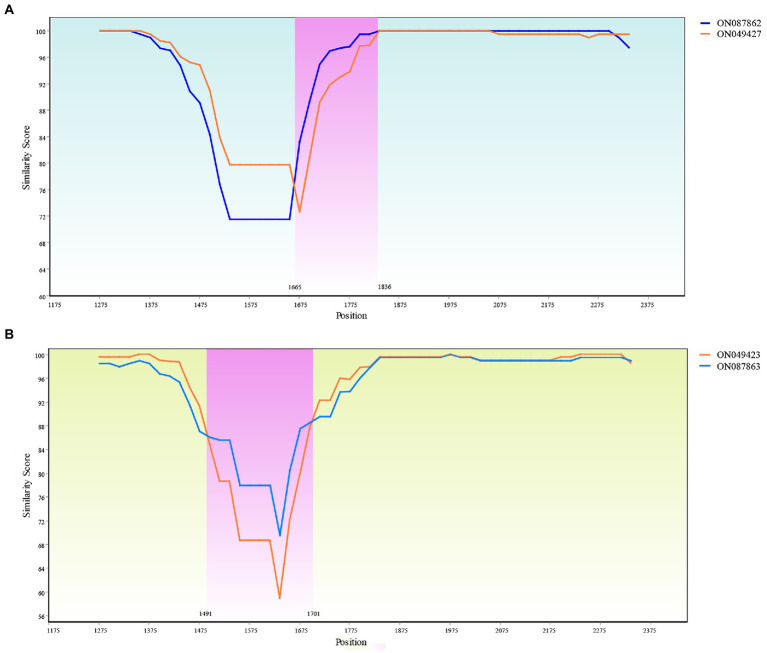
Recombination analysis of the ON045425 and ON087858. **(A)** Recombination of the ON045425. The recombinant main breakpoints were located at 1665 bp and 1836 bp. **(B)** Recombination of the ON087858. The recombinant main breakpoints were located at 1491 and 1701 bp.

Two recombination events were identified with one occurring between ON087862 and ON049427 to produce recombinant ON045425. A SimPlot of this recombination event is presented in Figure 10A, where the haplotypes ON049427 and ON087862 were used as the major and minor parental sequences, respectively. Another recombination event occurred between ON087863 and ON049423 to generate the recombinant ON087858. A SimPlot of this recombination event is presented in Figure 10B, in which the haplotypes ON087863 and ON049423 were used as the major and minor parental sequences, respectively.

## 4. Discussion

Based on the similar morphological features of the spores, and the high SSU rDNA sequence identity (Table 3), the microsporidium studied in present paper can be identified to be *P. alburnus* which was previously described from the liver of *C. alburnus* collected from Poyang Lake. However, it has been reported that the interspecific SSU rDNA sequence similarity could be high (up to 99%) among some fish-infecting microsporidian species, such as *Spraguea* spp. (Casal et al., 2012) and *Glugea* spp. (Mansour et al., 2020). Therefore, a question will be raised whether these 7 East Asian minnows-infecting microsporidians are conspecific or of different taxa. Intraspecific morphological plasticity has been intensively reported for many fish microsporidians, like *Ovipleistophora ovariae* (Weng et al., 2021a), *O. diplostomuri* (Weng et al., 2021a) and *Pleistophora hyphessobryconis* (Li et al., 2012). Although the spore size and spore wall thickness of *P. alburnus* in current isolates from the ovary differ from those in previous isolates from the liver, the internal structure characteristics of the spores are consistent, demonstrating that few intraspecific morphological variation among *P. alburnus* isolates was found here. The phenomenon may be caused by different infection tissue. Notably, all *P. alburnus* isolates herein infected solely the ovary, rather than the liver, which was one of the definitive taxonomic features of the genus *Pseudokabatana*. Indeed, the switch of infected tissue has been intensively reported for many microsporidians which were originally thought to be tissue specific. The genus *Ovipleistophora* was originally defined to be the obligate parasite of the fish ovary (Pekkarinen et al., 2002), however, Lovy and Friend (2017) found that *O. diplostomuri* could infect both the liver of bluegill sunfish *Lepomis macrochirus* and the cyst wall of the digenean parasite *Posthodiplostomum minimum*. Taken together, the tissue specificity inferred from the site of infection of type species was suggested not to be the reliable taxonomic criterion of the genus *Pseudokabatana.* Furthermore, it can be supposed that the ovary is the common infection site for *P. alburnus*, given that the sample size in the present study is far higher than that of previous report (Liu et al., 2019).

Ribosomal DNA loci are the most widely used molecular genetic marker for the species identification, the analysis of intraspecific variability, and phylogenetic relationship of microsporidia (Haro et al., 2003; Santin and Fayer, 2009; Wilkinson et al., 2011; Cordes et al., 2012; Li et al., 2012; Gonzalez-Tortuero et al., 2016). However, the existence of multiple ribosomal RNA gene copies in individual species, or even an individual spore has been reported in some insect-infecting microsporidia, such as *Nosema ceranae* (Sagastume et al., 2011) and *N. bombi* (Tay et al., 2005; O'Mahony et al., 2007), suggesting that the ribosomal DNA sequence is not a suitable molecular marker to discriminate their intraspecific variation. In the present study, we demonstrated the intraspecific variation of ITS sequences of *P. alburnus* and the conservative SSU and LSU rDNA sequences of *P. alburnus*. The intraspecific variation of ITS sequences of *P. alburnus* is similar with some fish-infecting microsporidia, such as, *Nucleospora salmonis* (Gresoviac et al., 2000) and *O. diplostomuri* (Weng et al., 2021a). Therefore, it can be suggested that SSU and LSU rDNA sequences are reliable molecular markers for the identification of *P. alburnus* and ITS rDNA sequence is appropriate to discriminate among their isolates.

The Rpb1 gene was also applied into the species classification and intraspecific variation of microsporidia (Gomez-Moracho et al., 2014; Maside et al., 2015; Kyei-Poku and Sokolova, 2017; Pretto et al., 2018; Tokarev et al., 2018, 2020; Yurakhno et al., 2021), which was generally thought to be of single copy and possesses more variable sites than those of the SSU rDNA, However, the amplification of Rpb1 is difficult and only a small amount of Rpb1 gene sequence is so far available in the public database. In the present study, failing to amplify Rpbl of some *P. alburnus* isolates with the same primer pairs and conditions can partially explain the problem. Based on the comparison of the obtained Rpb1 sequences, the intraspecific genetic variations of Rpb1 were remarkable among *P. alburnus* isolates and even in single host individuals. The similar findings were previously also reported in some insect-infecting microsporidia, such as *N. ceranae* (Gomez-Moracho et al., 2014), *N. apis* (Maside et al., 2015) and *N. bombycis* (Ironside, 2013). The coexistence of different Rpb1 sequences in an individual host was initially thought to be the result of co-infection of different isolates in the same host (Gomez-Moracho et al., 2014; Maside et al., 2015). Here, only one ITS rDNA haplotype of *P. alburnus* was detected within several single host individuals, suggesting that only one *P. alburnus* isolate was present in these individual host. The presence of intergenomic diversity in these species was one of the possible factors to explain it. Ironside (2013) found that the high genetic variation of protein-coding sequences is provided by intergenomic diversity of microsporidia within single hosts. The occurrence of recombination in *P. alburnus* further increases genetic diversity. Thus, the high genetic diversity of *P. alburnus* in single individual host could be due to the presence of intergenomic variation and recombination events. Given the existence of multiple Rpb1 haplotypes of *P. alburnus* in single individual host, we thought that Rpb1 is not a reliable marker to discriminate different *P. alburnus* strains.

Haplotype networks analyses indicated that ITS and Rpb1 haplotypes isolated from same location did not clearly cluster with the geographical locations, indicating that there is no geographical population divergence. The low *F*st indices and AMOVA analysis further supported it. The rapid geographical expansion of the parasite caused by their host dispersal among the investigated locations, especially after the construction of the South-to-North diversion project (Qu et al., 2020) is one of possible reasons for the low population differentiation among all *P. alburnus* isolates collected from the middle and lower reaches of the Yangtze river.

The occurrence of recombination between *P. alburnus* haplotypes indicates the possible cryptic sexual reproduction of this fish-infecting microsporidian. Sexual reproduction has previously been reported for microsporidia infecting daphnids (Haag et al., 2013; Gonzalez-Tortuero et al., 2016), insects (Sagastume et al., 2011; Wilkinson et al., 2011), and mammals (Wan et al., 2016). Ironside (2007) thought that the ancestor of the *Nosema*/*Vairimorpha* group was sexual and sexual process has been lost on multiple independent occasions during their evolutionary history. The genes associated with meiosis were also detected in *Edhazardia aedis*, *Vavraia culici* (Desjardins et al., 2015), and the early branching microsporidia, such as *Mitosporidium daphniae* (Haag et al., 2014) and *Amphiamblys* sp. (Mikhailov et al., 2017). Therefore, it can be supposed that meiosis and sexual reproduction may be common in Microsporidia, and sexual reproduction may be an ancestral feature of Microsporidia. The loss of sexual process in some species may be the result of habitat adaptation. Sex of the *Nosema*/*Vairimorpha* group has been lost on multiple independent occasions during their evolutionary history (Ironside, 2007). Interestingly, the all intra-fish developmental stages of *P. alburnus* were of isolated nuclei, and no diplokaryotic cells were observed, so it can be assumed that the possible invertebrate host of *P. alburnus* (supposed to be a freshwater shrimp) might involve the meiosis and sexual reproduction. *Inodosporus*, the closest branch relative of the genus *Pseudokabatana*, have been suggested to have a multi-host life cycle between fish and crustacean hosts where different developmental patterns occurred (Yanagida et al., 2023). The presence of diplokaryotic sporonts and uninucleate spores were observed within the-crustacean host developmental stages of *Inodosporus* species (Azevedo et al., 2000).

Although horizontal transmission is the most common transmission mode for fish-infecting microsporidia (Lom and Dyková, 2005; Phelps and Goodwin, 2008), vertical transmission has also been reported in some ovary-infecting microsporidian species, such as *O. ovariae* (Phelps and Goodwin, 2008) and *Pseudoloma neurophilia* (Sanders et al., 2013). In the current study, *P. alburnus* was generally found in fish ovary, implying that vertical transmission may exist in the *P. alburnus*. Vertical transmission may occur during spawning with contaminated spores from the infected ovary tissues. The possible transovum transmission of *P. alburnus* warrants further study.

In summary, we here firstly identified *P. alburnus* from the ovary of six East Asian minnows which extends its host range and geographical distribution. The intraspecific genetic variation was distinct among different *P. alburnus* isolates, but absence of remarkable population differentiation. The occurrence of recombination events among Rpb1 haplotypes indicates that sexual reproduction and multi-host life cycle exist for *P. alburnus*. In addition, we found that the liver was not the reliable taxonomic criteria of the genus *Pseudokabatana*, and proposed that fish ovary was be the general infection site of *P. alburnus*.

## Data availability statement

The datasets presented in this study can be found in online repositories. The names of the repository/repositories and accession number(s) can be found at: https://www.ncbi.nlm.nih.gov/genbank/, OM423155–OM423161; https://www.ncbi.nlm.nih.gov/genbank/, OP942336; https://www.ncbi.nlm.nih.gov/genbank/, OM423163-OM423170; https://www.ncbi.nlm.nih.gov/genbank/, OP948229; https://www.ncbi.nlm.nih.gov/genbank/, ON049422-ON049429; https://www.ncbi.nlm.nih.gov/genbank/, ON087855-ON087869; https://www.ncbi.nlm.nih.gov/genbank/, OP150918.

## Ethics statement

The animal study was reviewed and approved by The Animal Research Ethics Committee of the Institute of Hydrobiology, Chinese Academy of Sciences.

## Author contributions

MW collected the samples, performed the parasitological examination, data analysis, and helped write the manuscript. XZ, ZX, SX, QZ, and AL performed the morphological comparisons and helped write the manuscript. JZ designed this study and drafted the manuscript. All authors contributed to the article and approved the submitted version.

## Funding

This study was supported by the National Natural Science Foundation of China (32173019), Young experts of Taishan Scholars in Shandong Province (tsqn201909133), Initiative grant for high-level personnel recruitment in Qingdao Agricultural University, JSPS Bridge Fellowship (BR221304) awarded to JZ and the “First Class Fishery Discipline” programme in Shandong Province, China; the Talent plan “One Thing One Decision (Yishi Yiyi)” in Shandong Province, China.

## Conflict of interest

The authors declare that the research was conducted in the absence of any commercial or financial relationships that could be construed as a potential conflict of interest.

## Publisher’s note

All claims expressed in this article are solely those of the authors and do not necessarily represent those of their affiliated organizations, or those of the publisher, the editors and the reviewers. Any product that may be evaluated in this article, or claim that may be made by its manufacturer, is not guaranteed or endorsed by the publisher.

## Footnotes

^1^ http://tree.bio.ed.ac.uk/software/figtree/

^2^ https://www.fluxus-engineering.com/sharenet.htm
