# Supplementary figures and images for: Intraspecific genetic diversity of the fish-infecting microsporidian parasite Pseudokabatana alburnus (Microsporidia)
Source: Front Microbiol. 2023 Mar 9;14:1129136. doi: 10.3389/fmicb.2023.1129136 (PMC10034183; doi:10.3389/fmicb.2023.1129136)

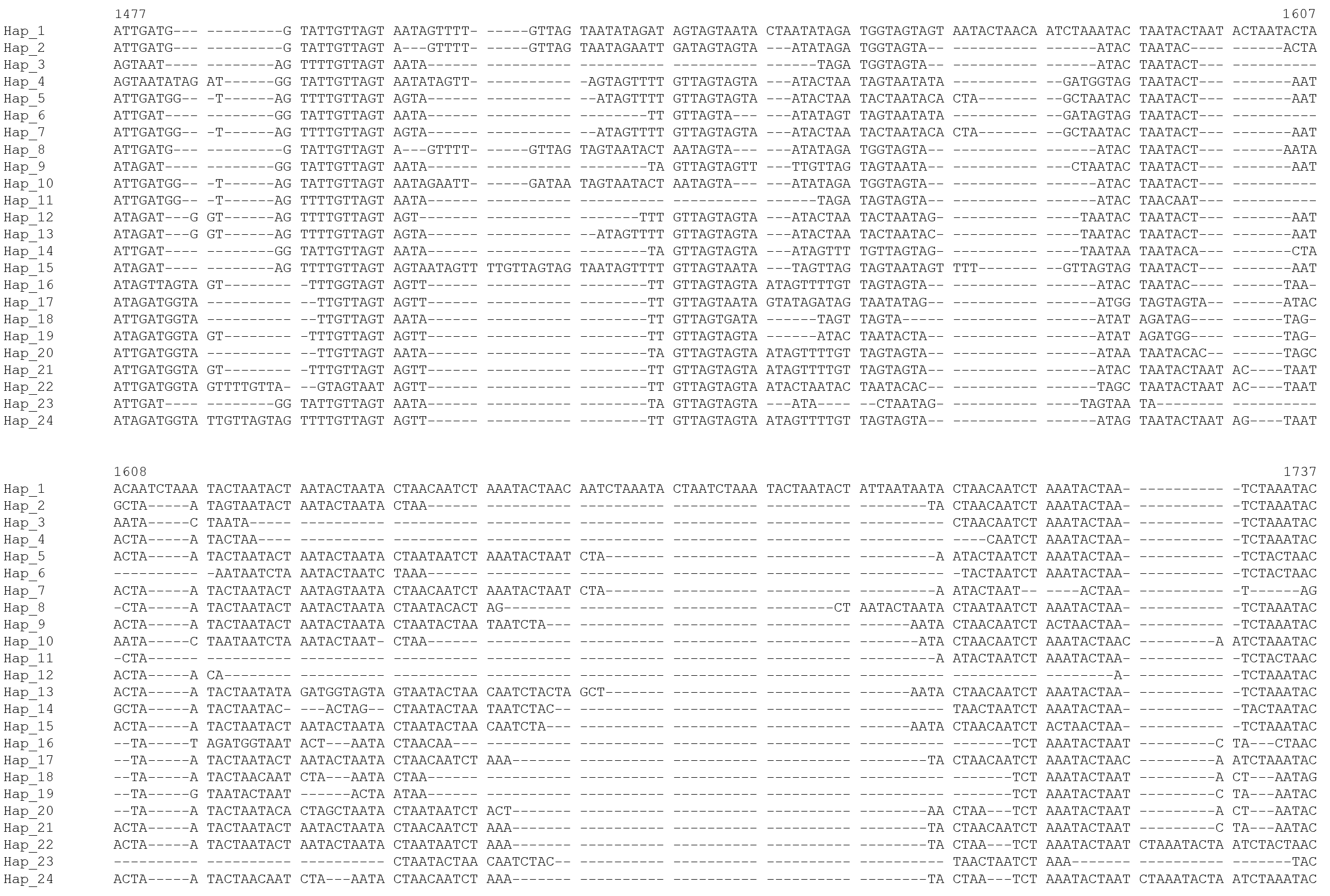

Supplement: Supplementary file 1 [file Image_1.TIF]
